# Long-term impact of undergraduate community-based clinical training on community healthcare practice in Japan: a cross-sectional study

**DOI:** 10.1186/s12909-020-02258-3

**Published:** 2020-10-01

**Authors:** Shinsuke Yahata, Taro Takeshima, Tsuneaki Kenzaka, Masanobu Okayama

**Affiliations:** 1 Division of Community Medicine and Medical Education, Kobe University Graduate School of Medicine, Kobe University Graduate School of Medicine, 2-1-5, Arata-cho, Hyogo-ku, Kobe, Hyogo 652-0032 Japan; 2 Department of General Medicine, Shirakawa Satellite for Teaching and Research (STAR), Fukushima Medical University, 2-1, Toyochikamiyajiro, Shirakawa, Fukushima, 961-0005 Japan; 3 Center for Innovative Research for Communities and Clinical Excellence (CIRC2LE), Fukushima Medical University, 1, Hikarigaoka, Fukushima, Fukushima 960-1295 Japan; 4 Division of Community Medicine and Career Development, Kobe University Graduate School of Medicine, Kobe University Graduate School of Medicine, 2-1-5, Arata-cho, Hyogo-ku, Kobe, Hyogo 652-0032 Japan

**Keywords:** Community-based medical education, Clinical training, Undergraduate, Community healthcare, Rural

## Abstract

**Background:**

Community-based medical education (CBME) has been evolving globally. However, the long-term impacts of CBME programs on career intention are ambiguous. Therefore, this study aimed to reveal the long-term impact of community-based clinical training (CBCT) such as CBME programs in Japan on current community healthcare (CH) practice.

**Methods:**

This cross-sectional study targeted physicians who had graduated from Kobe University School of Medicine between 1998 and 2004 and had over 15 years’ experience after graduation. Self-administered questionnaires were mailed to participants between September and November 2019. Of the 793 potential subjects, 325 questionnaires were undeliverable. A total of 468 questionnaires substantially sent to the subjects. The exposure was the undergraduate CBCT defined as clinical training about CH in a community. The primary outcome was the provision of current CH practice. The secondary outcome was rural retention. The odds ratios (ORs) and confidence intervals (CIs) were calculated, and the confounders (age, gender, and attitude toward CH at admission; primary outcome, and age, gender, attitude toward rural healthcare at admission, own and spouse’s hometown, and emphasis on child education; secondary outcomes) were adjusted using multivariate logistic regression analysis.

**Results:**

A total of 195 (41.7%) questionnaires were analyzed. The mean (standard deviation [SD]) age of study participants was 43.8 (3.5) years and 76.4% were men. A total of 48 physicians (24.6%) experienced CBCT, of which the mean (SD) training period was 26.3 (27.3) days. As many as 148 (76.3%) physicians provided CH at the time of the study, and 12 (6.5%) worked in rural areas. There was no notable impact of undergraduate CBCT on current CH practice (OR, 1.24; 95% CI, 0.53–3.08; adjusted OR [aOR], 1.00; 95% CI, 0.43–2.30) and rural retention (OR, 0.59; 95% CI, 0.06–2.94; aOR, 0.59; 95% CI, 0.11–3.04).

**Conclusions:**

It may be insufficient to use conventional CBCT in Japan to develop CH professionals effectively. Japanese CBME programs should be standardized through a review of their content and quality. They should continue to be evaluated for their medium- to long-term effects.

## Background

Community-based medical education (CBME), which is a style of education that places medical students into communities, has been evolving globally. It brings broad skills and ethical competences to medical students [1–3] and promotes their career intentions toward primary care [1, 2, 4, 5] and rural healthcare (RH) [1, 6, 7]. Aging populations have become an enormous challenge worldwide [8]. To deal with this problem, more attention is being focused on primary and community-based care [9]. Fostering healthcare professionals who provide primary care and community healthcare (CH) [10] is key to addressing this challenge. It is important to provide such care to various communities in each area, including rural ones. These social contexts emphasize the importance of CBME.

However, the long-term impacts of CBME programs are ambiguous. There are some limitations in the previous findings on career intention [1, 2, 4–7]. These studies have evaluated the changes in short-term career intentions or the effectiveness of meticulously designed special programs that include a strong selection bias during recruitment. There are some reports that rural origins and original intentions have stronger associations with career choices than the program itself [11, 12]. Therefore, the long-term impacts of CBME programs on their actual career choice that are adjusted to the participant’s origin and original intentions are controversial. If the long-term impact of an undergraduate CBME program on career choice for primary care and community healthcare is revealed, CBME can be promoted to foster healthcare professionals more evidentially.

CBME has also been evolving in Japan. The model core curriculum for medical education in Japan was formulated in 2001, and the CBME section was introduced in the revised edition that came out in 2007 [13]. A few Japanese reports have addressed the educational effects of CBME, and only the short-term effects of the previous reports have been observed [14–16]. However, Japanese medical universities have traditionally provided some medical students with the opportunity to practice at community hospitals and clinics. If these conventional CBME programs had shown long-term effectiveness, there would be little need to reconstruct the programs. If the programs did not work, they would need to be reconstructed. To construct CBME in Japan, an advanced aging country, it is necessary to confirm this ambiguity, and, to appropriately evaluate the long-term effects of Japanese conventional CBME programs.

This study aims to examine whether community-based clinical training (CBCT), such as CBME programs in Japan, has a long-term impact on the practice of CH for physicians by comparing whether CH was practiced as a professional career between physicians who had received CBCT during undergraduate study and those who had not.

## Methods

A cross-sectional study using an anonymous self-administered questionnaire was conducted to reveal the long-term impact of CBCT in Japan on career intentions toward CH.

### Participants

Physicians who graduated from Kobe University School of Medicine between 1998 and 2004, who had over 15 years’ experience after graduation, were included to evaluate the long-term effects of the undergraduate CBME program. We supposed that many physicians had made a stable career choice after about 10 years of post-graduate training. Thus, we selected 15 years in this study in order to evaluate the career intentions more substantially. In the past, the clinical training at Kobe University were the university hospital based clinical training by rotating each department of the hospital for one or two weeks. However, the medical students of Kobe University could take a two-week clinical training in the community hospitals or the clinics outside of Kobe University Hospital until three times. Thus, CH education at Kobe University was provided as an elective program. The training program in each training site depended on the practice providing by each community hospital or clinic. Therefore, the training programs were not standardized in aspect of quality and quantity. Participants in this study were undergraduates during this period, which is why some of them received community-based training. Thus, defining the CBCT, we conducted this study. Although CH education at Kobe University has been still an elective program, the standardized two-week CBCT programs as defined in this study has introduced into Kobe University in 2014.

We extracted the names and institutions from the graduate list and mailed questionnaires to institutions between September and November 2019. Reminders were mailed up to two additional times to subjects that did not respond within three to four weeks.

### Definitions

#### CH practice

The WHO Centre for Health Development defined CH as a comprehensive care approach integrating health and social services at the community level [17]. However, there were no prior studies that had specified practical items in detail regarding CH. Thus, based on the primary care commitment in the community [18] and considering the Japanese healthcare context, we defined CH practices as follows: (1) home care (home medical care and participation in discharge planning conferences [DPC]), (2) preventive care (vaccination, health education for residents or patients, and medical checkups), (3) interprofessional collaboration (with healthcare, welfare, and administrative professionals, with community residents, and participation in community care conferences [CCC]), (4) caring for the social security system (comprehension of long-term care insurance system and involvement in community-based integrated care systems), and (5) education for young professionals on CH (at primary care setting and educational institutions). In the Japanese context, DPC refers to an interprofessional meeting at the time of discharge, and pertains to coordinating care in a hospital, a living environment, and community health and social care services. The CCC is also an interprofessional meeting in which professionals such as care managers, social workers, and public health nurses discuss difficult cases and issues in the community. It should be held at least once a month in each municipality under the Long-Term Care Insurance Act of Japan.

#### CBCT

CBME refers to an education style that places students into communities. It is a rather wide concept. Thus, in this study, we defined CBCT based on the previous literatures [14] as clinical training of home medical care, home nursing care, outpatient day long-term care, long-term care facility, rehabilitation, medical checkups, vaccination, health education for residents or patients, mobile clinic as well as outpatient and inpatient care in the community.

#### Rural areas

The data from the Population Census of Japan were used to assess rurality [19, 20]. Japan had 1742 municipalities across 47 prefectures as of 2015. Municipalities, that is, cities, towns, and villages, are basic administrative geographic units. The 1742 municipalities were divided into quintiles according to population density: “quintile 1” with the lowest and “quintile 5” with the highest densities. The cut-off values for the quintiles were 42.8, 118.8, 308.8, and 1078.1 persons per square kilometer. Then, the municipalities of quintiles 1 to 3 were defined as “rural areas,” and the others were defined as “urban areas.”

### Measures

The questionnaire (available in Additional File 1) was developed through literature review and discussion between the co-researchers to improve its validity [21]. The questionnaire used in the previous study evaluating the short-term effects of the CBCT [14, 15] was adapted for the questionnaire of this study concerning the CBCT programs and the perceptions for CH and RH. Furthermore, reviewing the previous study, we defined the CH, the CBCT, and the rural areas described above to improve its validity. The primary outcome of this study was whether CH was currently being provided. We obtained the items related to CH using a 5-point Likert scale (i.e., “usually,” “often,” “sometimes,” “rarely,” or “never”). The responses “usually” and “often” were divided further into “yes;” “sometimes,” “rarely,” and “never” were treated as “no.” Then, we defined provider of CH practices if any one item was “yes,” and non-provider for the others. Selecting general medicine as the main specialty and rural retention were set as secondary outcomes.

The exposure of this study was whether the participant had experienced undergraduate CBCT. The item was obtained through a “yes” or “no” alternative question by looking back on participants’ past experience.

Participants’ age, gender, and attitude toward CH at the time of admission were considered as confounding variables. The attitudes toward CH were described as follows: “I think practicing CH is worthwhile” (“worthwhile”) and “I am confident of practicing CH” (“confidence”) [14]. While addressing the working area as the outcome, the following items were considered as confounding variables: age, gender, attitude toward RH at the time of admission, own hometown, spouse’s hometown, and emphasis on child education in choosing a living place. These items were chosen because they were listed as factors related to working area decisions by the WHO [22]. The attitudes toward RH were described as “I want to work in a rural area” (“rural”) [15]. These attitudes (i.e., “worthwhile,” “confidence,” and “rural”) were obtained using a visual analogue scale (VAS; 0 - 100 mm) and were treated as continuous variables. We assumed that subjective rural origin would affect personal choices more than actual rural origin. Thus, we obtained subjective values, that is, “urban,” “neither urban nor rural,” or “rural,” and each was treated as a dummy variable. The emphasis on child education was obtained with a 5-point Likert scale. “Yes” and “rather yes” were treated as “important,” and the others were treated as “not important.”

Although the timing of exposure and outcomes differ, all measures were obtained from this one-time questionnaire.

### Statistical analysis

First, we performed descriptive statistics. Groups with and without undergraduate CBCT were divided, and participants’ characteristics were analyzed with unpaired t-tests for continuous variables or chi-square tests for categorical variables. Following this analysis, we calculated crude odds ratios (ORs) and 95% confidence intervals (CIs) of exposure to outcomes as the main analysis, and then adjusted the ORs. We calculated the 95% CIs using logistic regression analysis. As part of a sensitivity analysis to treat unintended bias that may occur as a result of aggregating multiple items of CBCT and CH in one item, the effects of each CBCT item on current CH practice and the effects of CBCT on each current CH practice were evaluated using the same method of analysis. All statistical analyses were carried out using Stata MP version 15 (StataCorp, College Station, TX, USA).

### Sample size calculation

Power calculations were conducted to determine the sample size required to detect differences in providing the current CH between CBCT and non-CBCT groups. As there was no previous study that could be relied on for sample size estimation, we assumed that the proportion of CBCT was 0.1, the proportion of CH practice was 0.7 and 0.3 in the experience and control groups respectively, and was based on 80% power, setting alpha levels at *p* < 0.05 for statistical power calculation. The minimum completion sample size was calculated as 131 (CBCT group: *n* = 12, non-CBCT group: *n* = 119). The sample size was proposed as 772 assuming that 30% questionnaires could not reach the target population for various reasons such as not having address data on the graduate list or changed workplace. The response rate was 25%, and exclusion rate was 3%. There were about 100 Kobe University graduates each year. Thus, we registered physicians for 8 years. A total of 793 physicians who graduated between 1998 and 2005 were targeted in this study.

### Informed consent and reporting checklist

The questionnaire included explanations on participation, and participants were included in the study after checking whether informed consent had been obtained from them. This study was approved by the Institutional Review Board of Kobe University Graduate School of Medicine (number B190106). While writing this report, we used the STROBE cross-sectional checklist [23].

## Results

### Response rate

**Fig. 1:**
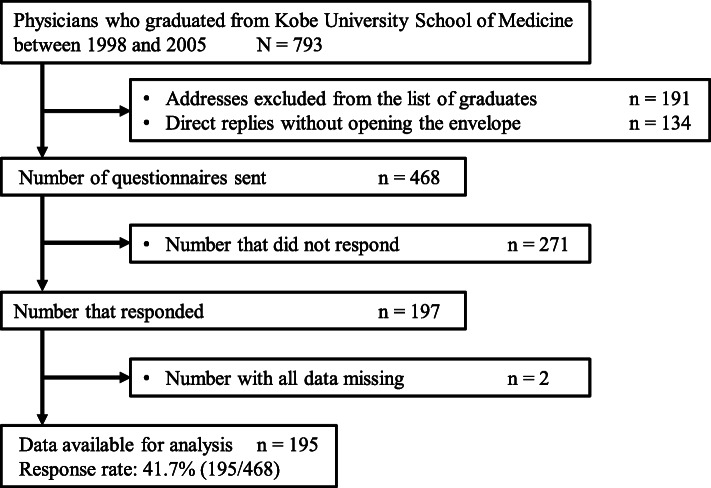
Flow chart with details of study participation

A total of 468 questionnaires (excluding 325 questionnaires that were undeliverable) were sent to the subjects, and 197 responses were received. All data were missing in two questionnaires, thus leaving 195 questionnaires for analysis. The response rate was 41.7% (195/468), see Fig. 1.

### Background and demographic factors

The mean (standard deviation [SD]) age of study participants was 43.8 (3.5) years and 76.4% were men. A total of 48 physicians (24.6%) had experienced CBCT. The mean (SD) training period of CBCT was 26.3 (27.3) days. All 23 known training spaces were located in urban areas. There seemed to be no apparent differences in the characteristics of the two groups. About three-quarters of the participants provided CH currently, only one selected general medicine as his main specialty, and twelve had worked in rural areas (Table 1; see the end of this text file). Based on these demographic statistics, a complete case analysis [24] was performed as the missing values for each variable were extremely small (i.e., from zero to four).

**Table 1:** Descriptive statistics of the study measures

	All	Experienced CBCT	Unexperienced CBCT	
	***n*** = 195	***n*** = 48	***n*** = 147	
	n (%)	n (%)	n (%)	***p***^**††**^
Age (year, mean, SD)^*^	43.8 (3.5)	43.1 (4.8)	44.0 (3.0)	0.11
Gender (male)	149 (76.4)	36 (75.0)	113 (76.9)	0.79
Attitude at admission				
“Worthwhile” (VAS; 0–100; mean, SD)^†^	37.8 (29.1)	42.5 (28.8)	36.3 (29.1)	0.21
“Confidence” (VAS; 0–100; mean, SD)^‡^	29.2 (24.8)	33.1 (25.7)	27.9 (24.4)	0.21
“Rural” (VAS; 0–100; mean, SD)^*^	21.7 (23.8)	22.3 (24.1)	21.5 (23.8)	0.83
Hometown				
Own (urban, neither, rural)	99 (50.8), 42 (21.5), 54 (27.7)	21 (43.8), 9 (18.8), 18 (37.5)	78 (53.1), 33 (22.5), 36 (24.5)	0.22
Spouse (urban, neither, rural)^*^	105 (54.1), 32 (16.5), 44 (22.7)	24 (50.0), 9 (18.8), 13 (27.1)	81 (55.5), 23 (15.8), 31 (21.2)	0.67
Emphasis on child education (important^a^)^*^	128 (66.0)	33 (68.8)	95 (65.1)	0.64
Details of CBCT				
Outpatient care		44 (91.7)		
Inpatient care		37 (77.1)		
Home medical care		19 (39.6)		
Home nursing care		8 (16.7)		
Outpatient day long-term care^*^		9 (19.2)		
Long-term care facility		10 (20.8)		
Rehabilitation		16 (33.3)		
Medical checkup		9 (18.8)		
Vaccination		11 (22.9)		
Health education for residents or patients		13 (27.1)		
Mobile clinic^*^		10 (21.3)		
Training period (day, mean, SD)^§^		26.3 (27.3)		
Rurality of the training site (rural)^॥^		0 (0.0)		
Current CH practices (provider^b^)^*^	148 (76.3)	38 (79.2)	110 (75.3)	0.59
Home medical care	10 (5.1)	3 (6.3)	7 (4.8)	0.69
Participation in discharge planning conference	41 (21.0)	10 (20.8)	31 (21.1)	0.97
Vaccination^†^	51 (26.4)	12 (25.0)	39 (26.9)	0.80
Health education for residents or patients^‡^	64 (33.3)	17 (35.4)	47 (32.6)	0.72
Medical checkup^†^	22 (11.4)	6 (12.5)	16 (11.0)	0.78
Collaboration with health professionals^‡^	24 (12.5)	5 (10.6)	19 (13.1)	0.66
Collaboration with welfare professionals^†^	33 (17.1)	8 (16.7)	25 (17.2)	0.93
Collaboration with administrative professionals^†^	20 (10.4)	5 (10.4)	15 (10.3)	0.99
Collaboration with community residents^¶^	17 (8.9)	4 (8.5)	13 (9.0)	0.91
Participation in community care conference^†^	26 (13.5)	7 (14.6)	19 (13.1)	0.80
Comprehension of long-term care insurance system^*^	70 (36.1)	17 (36.2)	53 (36.1)	0.99
Involvement in community-based integrated care system^*^	45 (23.2)	11 (23.4)	34 (23.1)	0.97
CH education at primary care setting	52 (26.7)	15 (31.3)	37 (25.2)	0.41
CH education at educational institution	44 (22.6)	9 (18.8)	35 (23.8)	0.47
Selecting general medicine^‡^	1 (0.5)	0 (0.0)	1 (0.7)	0.57
Working in rural area^**^	12 (6.5)	2 (4.4)	10 (7.1)	0.50

*Abbreviations*: *CBCT* community-based clinical training, *SD* standard deviation, “Worthwhile”, “I think practicing community healthcare is worthwhile”; “Confidence”, “I am confident about practicing community healthcare”; “Rural”, “I want to work in rural area”; *VAS* visual analogue scale, *CH* community healthcare

^*^ 1 person’s data were missing. ^†^ 2 person’s data were missing. ^‡^ 3 person’s data were missing. ^§^ 17 person’s data were missing. ^॥^ 23 person’s training places were identified. ^¶^ 4 person’s data were missing. ^**^ 9 person’s data were missing. ^††^ unpaired t-tests for continuous variables or chi-square tests for categorical variables

^a^ “Yes” or “rather yes” with 5-point Likert scale. ^b^ Any one item was “yes” in current CH practices

### Long-term impact of undergraduate CBCT on current CH practice

There was no notable impact of undergraduate CBCT on current CH practice (OR, 1.24; 95% CI, 0.53–3.08; adjusted OR [aOR], 1.00; 95% CI, 0.43–2.30) and rural retention (OR, 0.59; 95% CI, 0.06–2.94; aOR, 0.59; 95% CI, 0.11–3.04) (Table 2). The impact on selecting general medicine as the main specialty could not be analyzed owing to only one occurrence.

**Table 2:** Long-term impact of undergraduate community-based clinical training on current CH practice and rural retention

Outcome	OR (95% CI)	Adjusted OR (95% CI)
Current CH practice	1.24 (0.53 to 3.08)	1.00 (0.43 to 2.30)^a^
Rural retention	0.59 (0.06 to 2.94)	0.59 (0.11 to 3.04)^b^

*Abbreviations*: *CH* community healthcare, *OR* odds ratio, *CI* confidence interval

^a^ Adjusted for age, gender, and attitude toward CH at the time of admission (i.e., “I think practicing CH is worthwhile” and “I am confident of practicing CH”) using logistic regression analysis

^b^ Adjusted for age, gender, attitude toward rural healthcare at the time of admission, own and spouse’s place of origin, and emphasis on child education using logistic regression analysis

### Long-term impact of each experience on each current practice

There was also no notable relationship between CBCT and each current CH practice as well as between each CBCT and current CH practice (Tables 3 and 4). Clinical experiences of home medical care (OR, 1.74; 95% CI, 0.46–9.72; aOR, 1.57; 95% CI, 0.42–5.84) and long-term care facility (OR, 2.91; 95% CI, 0.38–130.48; aOR, 2.73; 95% CI, 0.32–23.55) seemed to have a minimal association with current CH practice.

**Table 3:** Long-term impact of each undergraduate community-based clinical training on current CH practice

Exposure	OR (95% CI)	Adjusted OR^a^ (95% CI)
Outpatient care	1.53 (0.62 to 4.13)	1.28 (0.52 to 3.12)
Inpatient care	0.96 (0.39 to 2.52)	0.79 (0.32 to 1.92)
Home medical care	1.74 (0.46 to 9.72)	1.57 (0.42 to 5.84)
Home nursing care	0.93 (0.16 to 9.74)	0.66 (0.12 to 3.65)
Outpatient day long-term care	1.09 (0.20 to 11.14)	0.85 (0.15 to 4.68)
Long-term care facility	2.91 (0.38 to 130.48)	2.73 (0.32 to 23.55)
Rehabilitation	1.38 (0.36 to 7.89)	1.01 (0.26 to 3.91)
Medical checkups	0.61 (0.12 to 3.91)	0.38 (0.08 to 1.77)
Vaccination	1.42 (0.28 to 14.01)	1.09 (0.22 to 5.57)
Health education for residents or patients	0.47 (0.13 to 1.93)	0.34 (0.10 to 1.20)
Mobile clinic	0.71 (0.15 to 4.45)	0.45 (0.10 to 1.99)

*Abbreviations*: *CH* community healthcare, *OR* odds ratio, *CI* confidence interval

^a^ Adjusted for age, gender, and attitude toward CH at the time of admission (i.e., “I think practicing CH is worthwhile” and “I am confident of practicing CH”) using logistic regression analysis

**Table 4:** Long-term impact of undergraduate community-based clinical training on each current CH practice

Outcome	OR (95% CI)	Adjusted OR^a^ (95% CI)
Home medical care	1.33 (0.21 to 6.14)	1.53 (0.30 to 7.80)
Participation in discharge planning conference	0.98 (0.39 to 2.30)	1.01 (0.44 to 2.33)
Vaccination	0.91 (0.39 to 2.01)	0.83 (0.37 to 1.89)
Health education for residents or patients	1.13 (0.53 to 2.36)	0.99 (0.48 to 2.03)
Medical checkups	1.15 (0.35 to 3.35)	1.48 (0.52 to 4.23)
Collaboration with healthcare professionals	0.79 (0.22 to 2.37)	0.58 (0.18 to 1.91)
Collaboration with welfare professionals	0.96 (0.35 to 2.42)	0.82 (0.31 to 2.16)
Collaboration with administrative professionals	1.01 (0.27 to 3.14)	0.73 (0.21 to 2.50)
Collaboration with community residents	0.94 (0.21 to 3.25)	1.06 (0.31 to 3.59)
Participation in community care conference	1.13 (0.37 to 3.07)	1.04 (0.36 to 2.97)
Comprehension of long-term care insurance system	1.01 (0.47 to 2.09)	0.95 (0.46 to 1.95)
Involvement in community-based integrated care system	1.02 (0.42 to 2.32)	0.97 (0.43 to 2.20)
CH education at primary care setting	1.35 (0.61 to 2.90)	1.35 (0.64 to 2.86)
CH education at educational institution	0.74 (0.29 to 1.75)	0.52 (0.21 to 1.27)

*Abbreviations*: *CH* community healthcare, *OR* odds ratio, *CI* confidence interval

^a^ Adjusted for age, gender, and attitude toward CH at the time of admission (i.e., “I think practicing CH is worthwhile” and “I am confident of practicing CH”) using logistic regression analysis

## Discussion

We found that there was no notable long-term impact of undergraduate CBCT on future CH practice. Nevertheless, clinical experiences of home medical care and long-term care facilities may have some association with the current CH practice.

### The impact of undergraduate CBCT on future CH practice

This study did not detect a positive relationship between the exposure of CBCT in undergraduate programs and the practice of CH in the future. There are some programs such as the Rural Physician Associate Program (RPAP) [25] or University of Missouri Rural Track Pipeline Program (MU-RTPP) [26] in the United States, and the Parallel Rural Community Curriculum (PRCC) [27] in Australia, which had long-term impacts on the specialty choices and places of practice. The findings in this study are inconsistent with those of these previous studies. We can refer to the four differences between these special programs and the CBCT of this study: participants’ characteristics, financial support, program location, and program style. Both these special programs and Kobe University CBCT were elective ones. However, while there were few differences between the participants and non-participants’ characteristics at Kobe University CBCT, there were explicit differences in the participants’ characteristics in other programs, particularly in terms of rural backgrounds and enthusiasm for rural practice. Furthermore, these special programs had substantial financial support in contrast to the Kobe University CBCT. They were carefully designed longitudinal integrated clerkships (LICs) and were mainly adopted in rural sites, while the Kobe University CBCT offered block-type clerkships, and was adopted in urban sites. Rural programs [7] and LICs [28] were reported to have an impact on career intentions. LICs immerses students in the community for a long period of 6 to 54 weeks [29]. However, participants in this study were provided only a 4-week mean training period. These differences may have resulted in inconsistent findings on the effects of undergraduate CBCT on providing CH practice in the future. While CBME is by itself effective, the programs that place medical students into the community may not be so.

### Insight for planning a CBME program

Although the main results of this study were negative, the sub-analysis provided some insights that were not statistically significant. However, the experiences of home medical care and long-term care facility had minimal impact on future CH practice. It is consistent with previous findings that a home care experience may motivate students toward CH [14]. Doctor-patient relationships and continuity of care [30] in home care experiences may affect students’ motivations. The reason why the experience of long-term care facilities affects students’ motivations toward CH is unknown, however it may promote awareness of the importance of integrated care that cannot be obtained with hospital-based training alone. These findings suggest that well-designed CBCT including these activities may have effect on engaging in CH practice in the future.

Certainly, the content and qualities of the Kobe University CBCT can depend on activities in each training facility. There may have been differences between the training years. However, we were not able to evaluate them sufficiently. The Kobe University CBCT was an elective program. Thus, it was not possible to evaluate the long-term effect of the undergraduate CBCT for all medical students. The Japanese government intends to support community healthcare professionals, especially physicians in rural areas, through development programs. To this end, the model core curriculum mandates participation in CBME for all medical students. The Japanese government has high expectations for CBME. It is clear that students’ characteristics and the rural training settings influence career choices. However, it is necessary to clarify the long-term effects of undergraduate CBCT on future CH practice for both medical students in general and students with specific characteristics (e.g., rural backgrounds, enthusiasm for rural practice, and scholarships). To generalize the positive effect of CBME on future CH practice, the conventional CBCT programs must be reconstructed and then be provided to all medical students. While constructing well-designed Japanese CBCT programs, incorporating experiences of home medical care and long-term care facilities may be effective. In order to evidentially promote CBME for fostering healthcare professionals, well-designed CBME programs should continue to be evaluated for both their short-term and medium- to long-term effects.

### Strengths of this study

This study evaluated the effectiveness of a CBCT conducted in a regular medical school. Although there are explicit efficacies of specific programs such as the RPAP and the PRCC, the CBME programs incorporated into standard medical school education are not sufficiently effective. Participants in this study were not given any financial support and were not restricted in any way. Thus, these results seem to have a higher degree of generalizability. These negative data suggest the appropriateness of reinforcing the conventional CBME programs. They also indicate the necessity of considering the influence of other factors, such as additional practices including residency programs, professional incentives, and experiences that affect personal preferences.

Furthermore, there is no evidence to present the long-term effects of the CBME program in Japan. Japanese CBME has a short history and is currently being developed. It is hoped that the long-term effects of the CBME program will be reported after this study. At that time, the results of this study will form important comparative data.

### Limitations

Kobe University CBCT was an elective program. Thus, those who chose the program may have already been motivated to practice CH. We adjusted the attitudes toward CH and RH at the time of admission, but the motivation level just before training could not be evaluated. If we had obtained information on the motivation just before training, it would have resulted in a measurement bias due to ambiguous memory. To avoid such a situation, attitudes at the time of admission were selected. Additionally, those currently practicing CH may be more likely to have a recall bias for CBCT. However, even if these biases were considered, the results did not show any difference, and the effect of biases seemed small. Also, response rate that was not so high was an issue. However, the proportion of male physicians who graduated from Kobe University between 1998 and 2004 was 72.0%, and the proportion of male respondents in this study was 76.4%. There might be not different, therefore, it suggested that the participants in this study were almost representative. Furthermore, there was only one participant who selected general medicine as their main specialty. Although the financial incentives of specialties other than general medicine are not great, Japanese physicians tend to prefer being specialists. In order to increase the number of physicians who specialize in general medicine, various efforts will be required. Finally, while this study evaluated the career choice of CH practice as the main outcome, CBME has many other implications [1–3]. At Kobe University, since the 2011 academic year (AY) following introduction of the CBME section in the 2007 model core curriculum, a community medicine course has been offered, which comprises lectures and workshops on CH. All medical students in their preclinical year have been offered the opportunity to practice at nursing homes since the 2015 AY and at special needs schools since the 2016 AY. Beginning with the 2017 AY, all medical students in their clinical year received visiting nursing practice. Although it is still not mandatory to provide medical students with training in rural programs and LICs, the significance of these community-based experiences should not be overlooked.

## Conclusions

The impact of the conventional CBCT in Japan on future practice of CH was not notable. However, the experiences of home medical care and long-term care facilities may be associated with providing CH practice in the future. The Japanese CBME has been providing for all medical students for a while now. However, it is currently being developed further. Therefore, a well-designed program that is able to effectively develop healthcare professionals toward becoming responsible for community healthcare would be necessary. It is also important to review the contents and qualities of the programs and to continue to evaluate its medium- to long-term effects.

## Supplementary information

**Additional file 1: Supplementary Table.** Question items in the self-administered questionnaire used in this study. Question items in the self-administered questionnaire used in this study.

## Data Availability

The datasets used and/or analyzed during the current study are available from the corresponding author on reasonable request.

## Abbreviations

CBME: Community-Based Medical Education
RH: Rural Healthcare
CH: Community Healthcare
CBCT: Community-Based Clinical Training
DPC: discharge planning conferences
CCC: community care conferences
VAS: Visual Analogue Scale
OR: Odds Ratio
aOR: Adjusted Odds Ratio
CI: Confidence Interval
SD: Standard Deviation
RPAP: Rural Physician Associate Program
MU-RTPP: University of Missouri Rural Track Pipeline Program
PRCC: Parallel Rural Community Curriculum
LIC: longitudinal integrated clerkship
AY: Academic Year
